# Coronary artery bypass grafting with concomitant resection for carcinoma of lung

**DOI:** 10.1016/S1674-8301(10)60013-9

**Published:** 2010-01

**Authors:** Yangyang Zhang, Yanhu Wu, Biao Yuan, Xiang Liu, Sheng Zhao, Zhi Li, Yu Xia

**Affiliations:** Department of Cardiothoracic Surgery, the First Affiliated Hospital of Nanjing Medical University, Nanjing 210029, Jiangsu Province, China

**Keywords:** lung cancer, coronary artery disease, lung resection, off-pump coronary artery bypass grafting

## Abstract

A 69-year-old woman with angina had a lesion in the left lower lobe on chest film. Angiography revealed coronary artery disease in three vessels. Combined off pump coronary artery bypass grafting (CABG) and left lower lobectomy were performed through median sternotomy. This approach avoids complications due to staged operations and cardiopulmonary bypass (CPB). This report shows that simultaneous off pump CABG and pulmonary operations can be performed safely in patients with coronary artery disease (CAD) associated with lung cancer.

## INTRODUCTION

Simultaneous surgical management of patients with coronary artery disease (CAD) and lung carcinoma still is a controversial issue. Rather than perform a pulmonary operation at the same time as a cardiac procedure, most surgeons would stage the surgical procedures, with the cardiac surgery operation first, followed by the pulmonary resection at a later period. This is because coagulation defects related to heparinization and inadequate exposure for lung resection are considered to increase the operative difficulty and mortality. However, delayed tumor resection may increase the risk of metastasis for the patient, increasing morbidity, mortality and cost. With the development of coronary artery surgery and endoscopic surgery, off-pump coronary artery bypass grafting (CABG) and co-existing pulmonary resection can be performed successfully and safely. This report presents our successful experience with concomitant off pump CABG and pulmonary resection for lung cancer.

## CASE REPORT

**Fig. 1:**
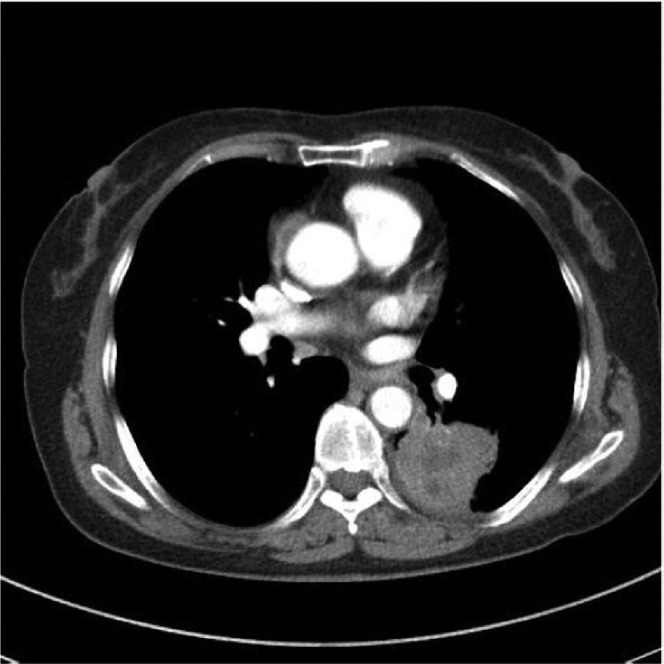
Computed tomography demonstrates a lesion at left lower lung.

**Fig. 2:**
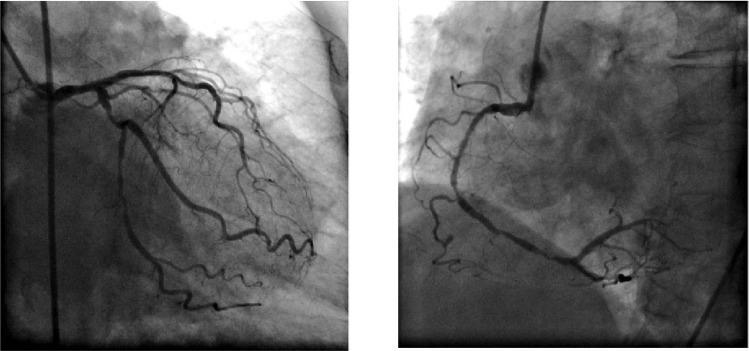
Cardiac catheterization demonstrates 20% stenosis of the left main coronary artery, 70% stenosis of the LAD and D1, 85% stenosis of the LCX, 90% stenosis of OM1, and 90% stenosis of the right coronary artery.

A 69-year-old woman was admitted with the clinical signs of coughing and hemoptysis for 3 months. Chest film showed a left lower lung mass. The patient reported good performance status before admission. Her physical examination was unremarkable. Pulmonary function testing revealed that her baseline forced vital capacity (FVC) and forced expiratory volume 1 s (FEV1) were 1.82 L and 1.61 L/s, respectively (68.5% and 72.2% of the predicted values, respectively). Contrast computerized tomography of the thorax demonstrated a 5.2×5.3 cm lesion in the left lower lobe (***Fig. 1***). Bronchoscopic examination revealed a hyperemic endobronchial tumor at the left posterior basal segment that yielded a diagnosis of adenocarcinoma. The ECG showed T waves changed in V3-6. Cardiac catheterization demonstrated 20% stenosis of the left main coronary artery, 70% stenosis of the LAD (left anterior descending) and D1(first diagonal branch), 85% stenosis of the LCX (left circumflex coronary artery), 90% stenosis of OM1(first obtuse marginal branch ), and 90% stenosis of the right coronary artery (***Fig. 2***).

Following general anesthesia, double lumen endotracheal intubation was performed. The patient was placed in a supine position. After midline sternotomy, the skeletonized left internal mammary artery (LIMA) was harvested. A long saphenous vein (SV) graft was simultaneously harvested. Anticoagulation was achieved using intravenous heparin (200 U/kg). The activated clotting time (ACT) was maintained above 300 s. The heart was stabilized using the Octopus suction stabiliser (Medtronic). Silastic intracoronary shunts were used routinely, and silastic “snares” were used selectively to encircle the target artery and provide proximal and/or distal control. The LIMA graft was anastomosed to the left anterior descending artery with continuous 7-0 polypropylene. A saphenous vein graft was grafted to the PDA (posterior descending branch) with a continuous suture. Another SV graft was sequentially grafted to OM1 and OM2 (second obtuse marginal branch). After completion of the distal anastomoses, the proximal SV graft anastomoses were performed on a disease-free segment of the aorta with the Novare Enclose II manual proximal anastomotic device. All proximal anastomoses were constructed manually with continuous 6-0 polypropylene.

After the CABG procedure, a lower left lung lobectomy with lymph node dissection was performed for the lung cancer. The grafted LIMA and anastomosed SV could be protected laterally from the operative field while performing the pulmonary resection via the midline sternotomy approach. The left lung was collapsed while the right lung was ventilated. The pulmonary ligament was divided to release the lung superiorly. The grafted LIMA and the anastomosed SV graft were protected by carbasus from the operative field. The lung parenchyma was retracted with conventional lung forceps. Additionally, the interlobar arteries were identified and dissected, using an endoscopic linear cutter. Silk sutures (3-0) were used to encircle and ligate the vessels. The pulmonary vein was managed identically to the interlobar artery. The interlobar bronchus was divided by an endoscopic linear cutter (45mm). When the inferior lobe was completely freed, it was retrieved and the lung was re-expanded to verify that the bronchial seal was adequate. Hilar lymph nodes dissection was performed, but incompletely. Two chest tubes were put in place, and the incision was closed in continuous layers. Postoperatively, the remaining left lung expanded without evidence of air leaks and the patient had an uneventful postoperative recovery. Final pathology revealed a 6cm moderately differentiated glandular carcinoma, and positive hilar lymph node with a free bronchial margin, pathological stage IIIA (T2N2). No complaints or clinical signs of recurrence were observed during the 5 month follow-up.

## DISCUSSION

Patients with lung carcinoma and coexisting CAD that is amenable to surgery are the best choice for the type operation described here. The surgical treatments for CAD and lung cancer are usually staged with the revascularization procedure occurring first, followed by the pulmonary resection at another time. The staged surgeries are feasible and safe, reducing postoperative morbidity and mortality. However this approach also has several disadvantages, such as the increased cost and discomfort of two separate operations. Furthermore, tumor growth and dissemination can occur during the interval between the two operations, which could worsen the prognosis. To resolve these problems, cardiovascular and thoracic surgeons have suggested simultaneous cardiac surgery with pulmonary resection as a safe procedure with a favorable outcome[1]–[5]. It was also reported that division of pulmonary fissures can be safely performed on a heparinized patient.

On the other hand, the altered immune response known to occur during cardiopulmonary bypass (CPB) at the time of cardiac surgery may also enhance the tumor growth and dissemination. Several authors have demonstrated that in patients undergoing combined surgery long-term survival is improved if the lung cancer is resected prior to CPB, compared with during CPB[1],[2],[5],[6]. Coronary artery bypass surgery without the CPB is an increasingly popular method that may be combined with other operations. It offers the advantages that it avoids the physiological stress associated with CPB (less pulmonary dysfunction, decreased incidence of postoperative renal failure) and the aortic manipulation that can lead to neurological injury through atherosclerostic embolisation. Off pump CABG reduces postoperative bleeding, transfusion requirements and hospital and/or ICU stay[7],[8]. In our case, after 16 hours ventilation, the patient was extubated with good arterial gases and stable hemodynamics. The blood loss of the 24 h postoperative period was 300 ml.

Median sternotomy allowed the cardiac and pulmonary surgery to be performed through a single incision. Compared with posterolateral thoracotomy, median stenotomy has been associated with a reduction in pulmonary dysfunction, analgesic requirements and postoperative pain[2],[9]. However, whether median sternotomy provides sufficient exposure for pulmonary lobectomy remains questionable. Left lower lobectomy was considered the most difficult pulmonary resection through a median sternotomy[1],[2],[5],[9]–[13]. The dissection tends to be obscured by the heart and retraction may induce cardiac arrhythmias and hemodynamic instability. However, the left lower lobectomy and complete hilar dissection was somewhat difficult for us to perform through the midline sternotomy. Opening the right pleura and pericardium widely, rotating the heart in an anticlockwise fashion and using the endocopic instruments can improve the exposure. Kanzaki et al [13] reported a case of a combined bilateral lung resection and off-pump coronary surgery though a midline sternotomy in a compromised lung function patient with both severe coronary artery disease and bilateral synchronous primary lung cancer. If the patient could tolerate the procedure, another simultaneous or staged left posterolateral thoracotomy would be possible.

Left thoracotomy is another choice, especially for patients with left sided lung cancer and CAD[11],[12],[14]–[17]. Badmanaban and colleagues[15] reported a 75-year-old patient with agina and a squamous carcinoma of the left lower lobe who underwent a single-stage procedure for the treatment of these lesions by a left posterolateral thoracotomy. Caimmi and Di Biasi[14] and Ahmed et al[12] considered that combined left lobectomy and off-pump CABG through left anterior thoracotomy was safe and effective. Ohtsuka and colleagues[18] successfully performed lingular segmentectomy and left anterior descending coronary artery bypass using video assisted limited anterior thoracotomy.

Combined operations involve other problems, such as potential bleeding related to heparinization, especially with CPB. Ulicny *et al* [19] reported bleeding problems in 15.8% of their patients. Morishita *et al* [1] also demonstrated that 2 of 6 patients required re-exploration due to bleeding. It is difficult to observe the bleeding in the pulmonary ligament through a median sternotomy. As a result it has been emphasized that pulmonary resection should be conducted after reversal of anticoagulation in simultaneous pulmonary surgery and off-pump coronary revascularization, and videoscopy has been recommended to check for suspected bleeding.

Based on our experiences, simultaneous off-pump CABG and pulmonary operations could be performed safety in a 69-year-old woman with CAD associated with left lower lung cancer. The one-step procedure facilitated the treatment of these two major problems, reducing hospitalization cost and resulting in acceptable outcomes.
